# A Cost-Minimization Analysis of a Medical Record-based, Store and Forward and Provider-to-provider Telemedicine Compared to Usual Care in Catalonia: More Agile and Efficient, Especially for Users

**DOI:** 10.3390/ijerph17062008

**Published:** 2020-03-18

**Authors:** Francesc López Seguí, Jordi Franch Parella, Xavier Gironès García, Jacobo Mendioroz Peña, Francesc García Cuyàs, Cristina Adroher Mas, Anna García-Altés, Josep Vidal-Alaball

**Affiliations:** 1 TIC Salut Social, Catalan Ministry of Health, 08005 Barcelona, Spain; francesc.lopez@cmail.cat; 2 CRES&CEXS, Pompeu Fabra University, 08003 Barcelona, Spain; 3 Faculty of Social Sciences, Universitat de Vic-Universitat Central de Catalunya, 08242 Manresa, Spain; jfranch@umanresa.cat (J.F.P.); XGirones@umanresa.cat (X.G.G.); 4 Health Promotion in Rural Areas Research Group, Gerència Territorial de la Catalunya Central, Institut Català de la Salut, 08272 Sant Fruitós de Bages, Spain; jmendioroz.cc.ics@gencat.cat; 5 Unitat de Suport a la Recerca de la Catalunya Central, Fundació Institut Universitari per a la recerca a l’Atenció Primària de Salut Jordi Gol i Gurina, 08272 Sant Fruitós de Bages, Spain; 6 Sant Joan de Déu Hospital, Catalan Ministry of Health, 08950 Barcelona, Spain; fgarciac@sjdhospitalbarcelona.org (F.G.C.); cadroher@sjdhospitalbarcelona.org (C.A.M.); 7 Agency for Healthcare Quality and Evaluation of Catalonia (AQuAS), Catalan Ministry of Health, 08003 Barcelona, Spain; agarciaaltes@gencat.cat

**Keywords:** cost analysis, health technology assessment, provider-to-provider telemedicine, telehealth, economic analysis

## Abstract

Background: Telemedicine (interconsultation between primary and hospital care teams) has been operating in the counties of Central Catalonia Bages, Moianès and Berguedà since 2011, specializing in teledermatology, teleulcers, teleophthalmology and teleaudiometries. For the period until the end of 2019, a total of 52,198 visits were recorded. Objective: To analyze the differential costs between telemedicine and usual care in a semi-urban environment. Methodology: A cost-minimization evaluation, including direct and indirect costs from a societal perspective, distinguishing healthcare and user’s costs, was carried out over a three-month period. Results: Telemedicine saved € 780,397 over the period analyzed. A differential cost favorable to telemedicine of about € 15 per visit was observed, with the patient being the largest beneficiary of this saving (by 85%) in terms of shorter waiting times and travel costs. From the healthcare system perspective, moving the time spent in a hospital care consultation to primary care is efficient in terms of the total time devoted per patient. In social terms and in this context, telemedicine is more efficient than usual care. Conclusion: Allowing users to save time in terms of consultation and travel is the main driver of interconsultation between primary and hospital care savings in a semi-urban context. The telemedicine service is also economically favorable for the healthcare system, enabling it to provide a more agile service, which also benefits healthcare professionals.

## 1. Introduction

Telemedicine nowadays coexists alongside conventional healthcare in most healthcare systems [1]. Although systematic reviews of its economic impact suggest that, for the time being, it is not suited to widespread implementation in all specialties and contexts [2,3], recent studies suggest that it is cost-effective in fields such as emergency medicine, cardiology, the management of diabetes and ophthalmology [4,5,6,7,8,9,10,11].

In Catalonia, the integration of the health information systems between primary care and specialized care allows for a fluid telemedicine-based case management. This implies relatively low coordination costs among different health specialties and incentivizes the use of these tools by health providers. Furthermore, the availability of information on healthcare activities provides an excellent opportunity to evaluate their impacts. To this end, this study case includes four telemedicine specialties (teledermatology, teleulcers, teleophthalmology and teleaudiometries) which are currently conducted in the Catalan public healthcare system, Central Catalonia Health Region. This includes the counties of Bages, Moianès and Berguedà, located in a large, mainly rural area, which also includes two major cities (Manresa and Berga) with an overall population of approximately 230,000 inhabitants.

A cost-minimization analysis performed in the same setting for the specific case of teledermatology [12] showed social savings of approximately €11.4 per visit, which have an impact, especially on users (77% of the total amount saved) as opposed to the healthcare system (23%). This is due to the size of the reduction in the commuting time and travel costs, which is especially significant in rural settings, a thesis which has been backed up by subsequent research [13]. Nevertheless, the study evaluated a short time period (teledermatology in 2016) and did not take into account other indirect costs such as the time spent by caregivers. In this context, the objective of the study is to broaden evidence on the economic impact of telemedicine with respect to usual care including other types of telemedicine (teleulcers, teleophthalmology and teleaudiometries) using a cost-minimization analysis from a societal perspective, including all feasible and significant direct and indirect costs.

## 2. Methodology

### 2.1. Service Description

**Figure 1:**
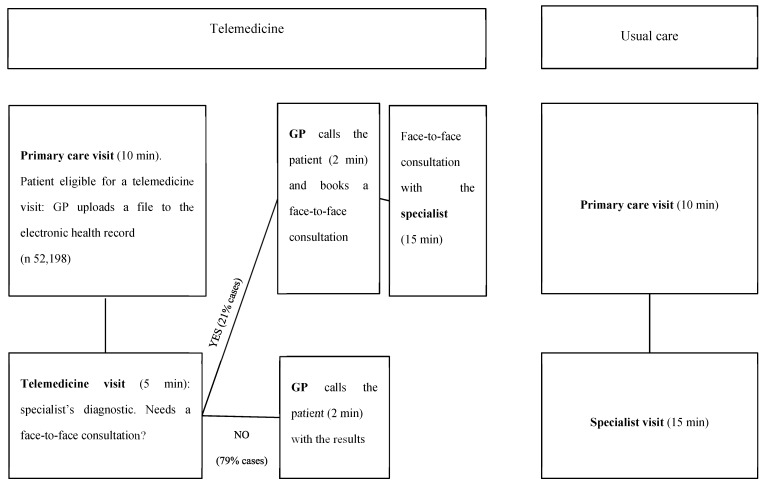
Patient flow: telemedicine vs. usual care.

The four studied telemedicine programs all operate in a similar manner: the primary care physician or nurse (salaried staff employed by the Catalan public healthcare system) uploads a file (such as a photograph) to the patient’s electronic health record together with their clinical notes; hospital specialists access the patient’s electronic health record, view the images and suggest treatment or an action plan; the primary care physician or nurse reviews the instructions and makes a phone call to the patient to give them the results of the consultation; if the specialist has any doubts, they can ask the primary care professional to arrange a face-to-face consultation with the patient (Figure 1). In other words, we can describe the process as medical record-based, store and forward and provider-to-provider asynchronous telemedicine between primary and hospital care. The Catalan healthcare system, which provides publicly financed universal health coverage, is free at the point of access, and thus, no fee is charged for the either face-to-face visits or the telemedicine service. We will assume that a telemedicine consultation avoids a face-to-face referral if it does not result in a referral for the same matter within the following 3 months. It has been shown that this telemedicine setting reduces waiting lists while improving access to GPs [14].

### 2.2. Study Type

A cost-minimization analysis was carried out over a three-month period using a societal perspective. Direct costs (healthcare costs corresponding to time spent by professionals and users during visits and travel expenses by users) and indirect costs (patient and caregiver’s time) were included. No staff training or equipment costs were included (practitioners used pre-existing devices), since they were not subject to the analyzed interventions. The cost estimate is based on 2019, a year which showed a higher number of telemedicine visits. A sensitivity analysis was carried out increasing the baseline costs. Calculations were performed using a Google Drive spreadsheet. The study was approved by the Ethical Committee for Clinical Research at the Foundation University Institute for Primary Health Care Research Jordi Gol i Gurina (registration number P19/182-P).

### 2.3. Direct Costs

The Catalan Institute of Health provided anonymized individual data regarding all 52,198 telemedicine consultation services performed during the period November 2011–November 2019. This dataset contains information on a case-by-case basis on the source and destination of every type of telemedicine service and whether it avoided a subsequent face-to-face visit or not. As Table 1 shows, all telemedicine services result in high face-to-face savings, ranging from 72% to 88% of the queries received.

In order to calculate the derived potential societal savings, differential costs attributable to the time spent by practitioners and citizens using telemedicine and usual care were taken into account. From the healthcare system point of view, the savings resulting from this form of intervention are based on the reduction of case management time. Whereas in usual care, the time spent on a face-to-face visit with a hospital care professional is 15 minutes, it is calculated that telematic monitoring of the case reduces the time to 5 minutes, redirecting the case back to the primary care professional, who calls the patient for approximately 2 minutes and closes the case, if applicable. If the specialist has any doubts, they can ask the primary care professional to book the patient for a face-to-face consultation (15 minutes). It was taken into account that, although in the teledermatology, teleophthalmology and teleaudiometry services, a primary care doctor is the one who makes the referral, in the case of teleulcers, a (primary and hospital care) nurse reviews the images and sends a reply. Baseline wages are used, according to standard labor agreements, for medical and nursing professionals in primary and hospital care. Travel costs (private car expenses) are calculated using the average travel distance (the methodology is described below) and the baseline price per kilometer.

### 2.4. Indirect Costs

**Figure 2:**
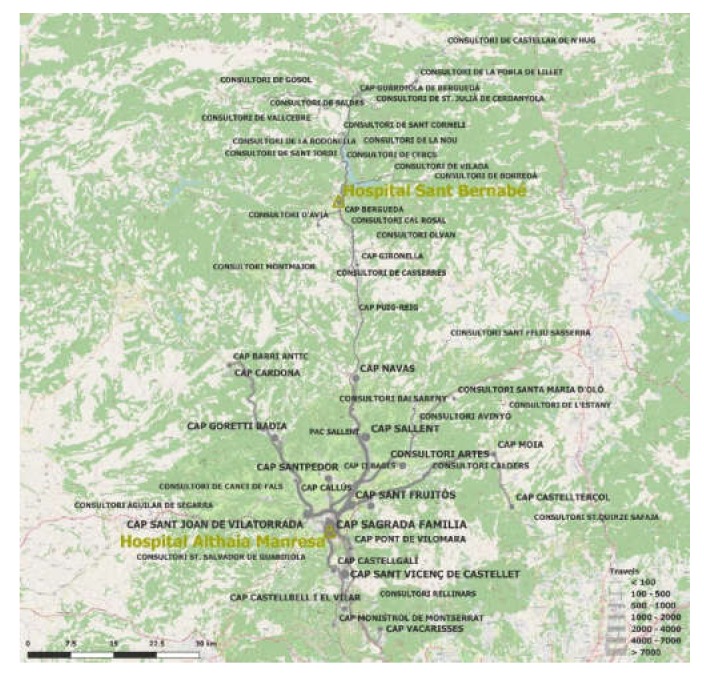
Origin and destination (either Hospital Sant Bernabé in Berga or Althaia Xarxa Assistencial Universitària in Manresa) of telemedicine visits avoided. The thickness of the line corresponds to the number of journeys saved.

Productive time (commuting to the hospital) lost by patients and caregivers was considered. The user also benefits from greater agility in the resolution of the case, reducing waiting time, as well as in terms of travel time to a hospital consultation (Hospitals in Manresa and Berga). Employing the methodology used by Vidal-Alaball et al. 2019 [13], through a combination of the R 3.6.1 software (The R Foundation for Statistical Computing, Vienna, Austria), a Google Maps API and the information from each of the user’s Primary Care Team (as a proxy for the user’s place of residence), together with the referral hospital, a very accurate calculation of the total number of kilometers and time of journeys saved by the intervention was obtained (Figure 2). Therefore, the sample saved 893,820 kilometers (21.58 km per case, for the round trip) and 16,812 hours (25 minutes per case) of travel. The costs to users (patient and caregiver) have been calculated by multiplying travel and consultation time by the average salary/hour.

Also, according to an aggregate analysis of the users’ profiles, it can be observed that the average age of a telemedicine service user is 52, with a standard deviation of 23, suggesting the heterogeneity of the beneficiary profile. If we assume that people aged over 65 (34% of the total sample) and under 16 (8%) require the company of a caregiver during their visits, this means that we have to add the indirect impact in terms of opportunity costs of the time spent by caregivers in 42% of the cases analyzed.

The nature of each type of cost is shown in Table 2.

Finally, Table 3 shows the parameters which were considered when making calculations and their corresponding sources: the hourly wages of professionals, the price per kilometer, the opportunity cost of the user, the total number of visits (saved), consultation time with the specialist with and without telemedicine, the primary care professional’s phone call time, and average time and distance for users. The results are shown for both perspectives (i.e., healthcare system and user).

## 3. Results

Table 4 shows the results of calculating the societal savings (distinguishing between those of the healthcare system and of the users) from the use of telemedicine in comparison to usual care. While the cost of making phone calls is exclusive to the telemedicine program (€ 42,675), there is a reduction in the time spent by hospital staff. Despite the fact that 21% result in a face-to-face visit, and that the salary per hour is higher in the context of primary care than in hospital care, the consultation time of 79% of cases was reduced by 8 minutes, implying savings in relation to usual care, where all visits are face to face and 15 minutes, for a professional time equivalent to € 154, 542 for the sample under analysis.

Regarding patients, while also taking into account cases where telemedicine is ineffective in avoiding a face-to-face visit, there is a saving on consultation and travel time of € 185,325 and € 317,995 respectively. These two parameters take into account the assumption that 42% of the cases had to be accompanied in face-to-face visits. In terms of fuel, the difference between the cost of telemedicine and usual care is € 165,211, calculated as the result of subtracting the product of the average travel distance per case (21 km) by the cost/km (€ 0.25) by the number of cases that have avoided a face-to-face visit (41,402, totaling € 223,455) and the equivalent cost from telemedicine visits that have not avoided a face-to-face visit (€ 58,244). Thus, the total of the costs and differential savings for the different types of telemedicine is approximately € 780,397 (a saving of € 15 per visit).

### Sensitivity Analysis: An Even More Favorable Scenario for Telemedicine

In order to comparatively evaluate the results, a maximum estimate of the sensitivity analysis is included by varying some of the assumptions (Table 5). This second scenario increases the costs included in Table 3 by 20%: the patient travel time and that of their possible companion (assuming that the actual time is not wholly shown in Google Maps, but that there are transaction costs derived from going to pick up the car, looking for a parking space, attending the consultation or waiting for the patient’s turn), the travel cost (measured in €/km, assuming that it could be increased with respect to the evaluation performed for teledermatology [12]) and the hourly wages of medical professionals (assuming that the real cost may be closer to the company cost, rather than the actual remuneration received by the health professionals). The results of this scenario show that the savings increase by approximately 8%, i.e., as much as € 17 per visit and continue to be mostly favorable for the user (85%). A sensitivity analysis was not performed for the opposite scenario, assuming that the calculation of time and distance savings made using Google Maps is, in itself, the minimum.

## 4. Discussion

### 4.1. In Relation to the Study with 2016 Data

The study concludes that, in the given context, telemedicine is an unequivocally preferable option to usual care from an economic point of view. The strength of this diagnosis is similar to that derived from the analysis performed with 2016 data for the specific case of teledermatology, i.e., the result of including other specialties (teleulcers, teleophthalmology and teleaudiometries), lengthening the time period (by using the complete sample available) and adding the indirect cost approach of the caregiver results in savings per visit 35% above the base case studied by Vidal Alaball et al. with 2016 data [12] (Table 6). We note that once caregivers’ opportunity costs are introduced, the most important differential corresponds precisely to the calculation of the cost in terms of the time of users. The similarity of results between the different types of costs reflects the robustness of the methodology used.

### 4.2. Sensitive Variables

The magnitude of the result is highly sensitive to the parameter corresponding to the opportunity cost (lost productivity) of the user and this has been calculated homogeneously among the different beneficiary profiles (minors, of working-age and retirees); although an eventual differential calculation by profile would not change the results, it would far better approximate the representative total of the savings. It should be borne in mind that in contexts with higher labor productivity of both professionals and users, the results of the analysis would be much more favorable to telemedicine.

With regard to the extrapolation of these conclusions and with the “travel time” factor, it is worth keeping in mind that the study was performed in a mostly rural and semirural setting. The average distance per journey may be higher than in urban settings, although it is not clear if the journey time would be higher (as moving within a city is much slower). Whatever the case, the results show that both factors (i.e., travel cost and time lost) are sufficient to reach the same conclusion, namely, that even if telemedicine did not save on travel costs (being “zero kilometer”), it would be cost-effective, and even if it did not save anything in terms of time (for the user and the healthcare system), it would also be cost-effective.

As to the assumption that patients travel by car, it is reasonable to assume that some of them use public transport. If we consider this possibility, telemedicine savings would be even higher, since in rural settings, where the frequency of public transport is very low, the potential savings in terms of travel costs (using public transport instead of private transport) would clearly be far outweighed by more travel time (with and without waiting time). In the context involved in the study, which was almost devoid of a railway network (except in the south of the city of Manresa), it is unlikely that the bus is faster than private transport.

### 4.3. Factors not Included in the Analysis

While it is true that this assessment includes the differential essential elements between the two analyzed models, it does not include objective or easily monetizable intangible factors such as the users’ and professionals’ satisfaction with the service or the improved management of cases in function of their clinical severity. This improvement in care management could reduce waiting lists to the access of GPs, one of the biggest problems in the Catalan healthcare system. In this context, telemedicine allows for better allocation of care time according to the complexity of the case. Future lines of research ought to quantify these factors, which are complementary but key in order to evaluate the service’s effectiveness.

In addition, the type of analysis performed assumes that clinical effectiveness is equivalent. Although a time period which includes aspects strictly related to management seems sufficient to make a good diagnosis, as is the case, and despite the complexity of the information which would be needed, we ought to try to ensure the hypothesis of equivalence in health impact and add any significant and differential costs which go beyond and which can be calculated in a rigorous manner.

It needs to be borne in mind that as doctors are remunerated, their increased productivity does not imply a direct translation into the healthcare provider’s income account; instead, the freer the practitioners, the fewer practitioners the healthcare provider will need to hire. In other words, savings might occur in the mid-term, as opposed to the short term.

It should also be considered that the increased ease with which referrals can be made might have incentivized GPs to use interconsultation as a second opinion tool to support the diagnosis of patients they would normally have treated. This might have increased the ratio of saved face-to-face visits.

Finally, it should be mentioned that the study also assumed that the differential cost of expenses such as cameras or clinical software is zero, since this was the case, but in the case of introducing this service from scratch in another context, these costs would have to be taken into account. In any case, the magnitude of the savings made by the service makes it unlikely that including them could significantly alter the results of the analysis.

## 5. Conclusions

The results show that telemedicine minimizes the costs of the two agents included in the analysis (i.e., the user and the healthcare system); from either perspective, telemedicine is better than usual care from an economic point of view. However, it was observed that from the € 14.95 saving per visit, approximately 85% benefits the patient, showing that this kind of intervention is especially convenient for the user, particularly for the time saving which it offers.

## Figures and Tables

**Table 1:** Number of telemedicine visits and% of face-to-face visits saved, per type.

Type of Telemedicine	Number of Visits	Face-to-Face Visits Saved (%)
Teledermatology	40,658	77.7
Teleophthalmology	1180	72.1
Teleaudiometries	9823	86.2
Teleulcers	537	88.5
Total (weighted average)	52,198	(79.3)

**Table 2:** Direct and indirect costs, for users and for the healthcare system.

	Direct Costs	Indirect Costs
**Users**	Travel costs	Time spent by caregiversTravel time
**Healthcare system**	GP’s timeNurse’s time	

**Table 3:** Calculation parameters.

	Concept	Amount	Source
**Costs (€)**	Wage/h primary care doctor	24.60	ICS [15]
	Wage/h primary care nurse	17.68	
	Wage/h hospital doctor	22.46	UCH [16]
	Wage/h hospital nurse	16.53	
	Travel cost per km	0.25 *	Own
	Average time value (patient and caregiver)	13.36	SAIT [17]
**Variables observed**	Total number of visits	52,198	Own
	Number of visits saved	41,402	
	Teleulcers number of visits	537	
	Teleulcers number of visits saved	472	
	Not teleulcers number of visits	51,661	
	Not teleulcers number of visits saved	40,930	
	Minutes with specialist in face-to-face visit	15 *	
	Minutes with specialist in teleconsultation	5 *	
	Minutes in primary care visit	2 *	
	Average travel distance km	21.58	R + Google API
	Average travel time	0.4	R + Google API

* For the comparability between studies, the baseline scenario takes the same parameter as in Vidal-Alaball et al. [12].

**Table 4:** Differential costs between telemedicine and usual care (in €).

	Concept	Telemedicine	Usual Care	Difference
**Healthcare system’s costs**	Primary care staff phone call	42,675		42,675
	Hospital staff	137,805	292,347	−154,542
**Users’ costs(patient and caregiver)**	Consultation time	62,240	247,565	−185,325
	Travel time	962	318,957	−317,995
	Travel cost (private car)	58,244	223,455	−165,211
	**Total**	301,926	1082,324	−780,397
	**Total per patient**	5.78	20.73	−14.95

**Table 5:** Sensitivity analysis: 20% increase in costs. Main results (€).

	Concept	Telemedicine	Usual Care	Difference
**Healthcare system costs**	Primary care staff phone call	51,210		51,210
	Hospital staff	165,366	350,816	−185,451
**User’s costs**	Patient: consultation time	50,221	247,565	−197,344
	Patient: travel time	1154	382,748	−381,594
	Travel cost (private car)	69,893	268,146	−198,253
	**Total**	337,844	1249,275	−911,431
	**Total per patient**	6.47	23,93	−17.46

**Table 6:** Differential costs per visit. Comparison between studies.

Type of Costs		Previous Study [12] (€)	Baseline Scenario (€)	Previous Study [12] (% of total)	Baseline Scenario(% of total)
**Healthcare system costs**	Primary care staff	0.77	0.82	22.60	14.33
	Hospital staff	−3.42	−2.96		
**User’s costs**	Time	−6.31	−9.64	77.40	85.67
	Travel cost	−2.76	−3.17		
**Total**		−11.71	−14.95	100	100
